# Retraction: Organometallic Ag nanostructures prepared using *Hypericum perforatum* extract are highly effective against multidrug-resistant bacteria

**DOI:** 10.1039/c9ra90026h

**Published:** 2019-04-12

**Authors:** Qaisar Maqbool, Dariusz Kruszka, Piotr Kachlicki, Gregory Franklin

**Affiliations:** Institute of Plant Genetics of the Polish Academy of Sciences 34 Strzeszynska Street 60-479 Poznan Poland fgre@igr.poznan.pl

## Abstract

Retraction of ‘Organometallic Ag nanostructures prepared using *Hypericum perforatum* extract are highly effective against multidrug-resistant bacteria’ by Qaisar Maqbool *et al., RSC Adv.*, 2018, **8**, 30562–30572.

We, the named authors, wholly retract this *RSC Advances* article.

The authors have repeated the FTIR and TGA analyses of the nanoparticles synthesised using *H. perforatum*, and also obtained the original data files from the facility that carried out the initial analyses. The authors have compared the new results and the original data files with the data in the published article. They found that the graphs presented in Fig. 7a and b in the article did not correspond to neither the original data nor the new analysis. They also found that the same TGA graph (Fig. 7b) was used in previous articles by the first author but representing a different material.^1,2^

The Institute of Plant Genetics of the Polish Academy of Sciences have confirmed to the Royal Society of Chemistry that graphs presented in Fig. 7 of this *RSC Advances* article were manipulated.

The authors would like to apologise for any inconvenience caused to readers.

Signed: Dariusz Kruszka, Piotr Kachlicki and Gregory Franklin

Date: 5^th^ April 2019

Qaisar Maqbool opposes this retraction.

Retraction endorsed by Andrew Shore, Executive Editor, *RSC Advances*

## Supplementary Material
